# Immuno-phage synergy driven by fitness costs: turning bacterial phage resistance into therapeutic gain through evolutionary traps

**DOI:** 10.3389/fimmu.2026.1856088

**Published:** 2026-07-01

**Authors:** Jumpei Fujiki, Asahi Yamada, Rin Shinzato, Rui Hiya, Nanako Norihisa, Kimita Imai, Emiru Wakabayashi, Satoshi Gondaira, Hidetoshi Higuchi, Hidetomo Iwano

**Affiliations:** 1 Laboratory of Veterinary Biochemistry, Department of Veterinary Medicine, Rakuno Gakuen University, Ebetsu, Hokkaido, Japan; 2 Animal Health Unit, Department of Veterinary Science, School of Veterinary Medicine, Rakuno Gakuen University, Ebetsu, Hokkaido, Japan; 3 Phage Therapy Institute, Comprehensive Research Organization, Waseda University, Tokyo, Japan

**Keywords:** antimicrobial resistance, bacteriophage, evolved phages, infection control, phage resistance

## Abstract

The host immune system typically recognizes administered bacteriophages as foreign entities, potentially acting as a rate-limiting factor in phage therapy via neutralizing antibody production and immune-mediated clearance. Furthermore, the emergence of bacterial phage resistance during treatment remains a major hurdle for its clinical application. Although employing phage cocktails is the standard strategy to suppress phage resistance, clinical trials occasionally report the emergence of phage-resistant variants despite cocktail treatments. Therefore, a more proactive strategic approach that goes beyond merely preventing resistance and instead actively steers the trajectory of bacterial evolution must be applied. In this context, immuno-phage synergy, in which phages and host immunity act in an orchestrated manner rather than in competition, has recently gained significant traction. To evade phage infection, pathogenic bacteria often mutate or shed crucial surface structures (e.g., capsules, lipopolysaccharides, and pili), thereby incurring fitness costs as evolutionary trade-offs. This mini-review summarizes recent insights into how these trade-offs drastically enhance host immune clearance, including increased susceptibility to neutrophil-mediated opsonophagocytosis, complement-dependent killing, and host chemical barriers. By understanding and exploiting the dynamics of phage-induced selective pressure and immunological trade-offs, the evolutionary arms race could be altered to intentionally drive pathogens into evolutionary traps. Concurrently, we discuss emerging clinical challenges, such as unexpected immune evasion (trade-up) resulting from certain mutations, and the critical disconnect between *in vitro* resistance assays and *in vivo* evolution. Ultimately, we highlight how the formidable barrier of phage resistance can be converted into a therapeutic gain, thereby offering a robust framework for sustainable phage therapy.

## 1 Introduction

Driven by the global public health crisis of antimicrobial resistance (AMR), the re-evaluation of bacteriophage (phage) therapy is rapidly advancing (1, 2). While recent clinical trials and the accumulation of compassionate use cases, particularly in Western countries, have demonstrated the efficacy of this therapy (3, 4), its fundamental challenges are by no means new. Indeed, early medical literature from nearly a century ago had already pointed out the existence of evidence for and against the therapeutic usefulness of phages and the contradictory literature surrounding them (5). Today, re-examining these historical challenges through the use of modern molecular biology and advanced analytical technologies allows us to clearly define several major barriers hindering the clinical success of phage therapy. Specifically, these are: (i) accessibility to the local site of infection, (ii) maintenance of phage titers, (iii) induction of anti-phage immune responses, including neutralizing antibodies, and (iv) the emergence of bacterial phage resistance.

The first three of these challenges can be controlled or mitigated to some extent through clinical interventions, such as the appropriate selection of target diseases and the optimization of administration routes and dosages. For instance, actual clinical cases suggest that the impact of neutralizing antibody production can be clinically overcome by selecting localized administration or adjusting dosing schedules (6–8). However, as long as phages are administered into a living organism to attack bacteria, there exists an unavoidable biological barrier: the “evolutionary arms race” between the viral predator and the bacterial host; namely, the evolution of phage resistance (4). Generally, the diverse phage defense systems possessed by bacteria dictate fundamental differences in susceptibility prior to treatment; however, much of the resistance induced during therapeutic intervention is driven by spontaneous mutations in molecules that serve as phage receptors (1, 4). The acquisition of such resistance mutations and the subsequent bacterial regrowth during treatment represent a major threat that directly impacts the clinical efficacy of phage therapy.

Currently, the use of phage cocktails, or mixtures of phages, is considered a fundamental principle for controlling this resistance. In particular, rational cocktail designs combining phages that recognize distinct receptors, such as lipopolysaccharides (LPS) and pili, have been advocated for multiple pathogen species (9, 10). In actual clinical settings, however, several cases have reported the emergence of multi-phage-resistant variants despite such optimal cocktail treatments (4, 11). This strongly suggests that modern phage therapy requires a more proactive strategic approach that goes beyond merely suppressing resistance through cocktails. One concept opening new avenues for phage therapy is that of bacterial fitness costs associated with phage resistance; that is, the loss of virulence or survival capacity incurred as a trade-off for mutating or shedding phage receptors (4, 12, 13).

In this context, this mini-review focuses on the relationship between the bacterial acquisition of phage resistance and the host immune response. By elucidating how the evolutionary trade-offs of phage resistance render bacteria vulnerable to the immune system, referred to as immuno-phage synergy, and using selective pressure to intentionally drive pathogens into “evolutionary traps”, we discuss how the formidable challenge of phage resistance can be converted into a clinical therapeutic gain.

## 2 The cost of phage resistance -bacterial perspective

The initial and crucial step of the phage lytic cycle is the adsorption of the viral particle to specific receptors on the bacterial surface (1, 2). Pathogenic bacteria display a diverse array of surface structures, such as LPS, capsular polysaccharides, wall teichoic acids, outer membrane proteins (OMPs), and appendages like pili and flagella, that inadvertently serve as phage receptors (14, 15). Consequently, a common and effective bacterial strategy to evade phage infection is the mutation, downregulation, or complete deletion of these receptor molecules. However, as dictated by the principles of evolutionary biology, such adaptation rarely comes without a price. In the perpetual “evolutionary arms race” between phages and bacteria, the acquisition of phage resistance through receptor modification can impose a fitness cost on the pathogen. It is crucial to recognize that these surface structures are not merely viral attachment sites; they are indispensable virulence factors required for establishing infection, host colonization, and maintaining bacterial integrity. Altering these structures to escape phage predation frequently disrupts their original biological functions, forcing a fitness trade-off. From an immunological perspective, the loss or truncation of these structures can create a critical vulnerability for the bacterium. When bacteria shed these critical outer layers to survive a localized phage attack, they concurrently disarm their own defense mechanisms against the host. In essence, just as pathogens successfully evade the viral threat by deploying phage resistance, they leave themselves susceptible to a devastating counterattack from the host immune system. This biological cost lays the vital mechanistic groundwork for the immuno-phage synergy discussed in the following section and summarized in **Table 1**.

**Table 1:** Recent representative examples of immuno-phage synergy and evasion driven by bacterial surface modifications.

Type	Category	Bacterial pathogens	Targeted phage receptor	Potential altered surface structure leading phage resistance	Expected immunological trade-offs/ups	Reference
Trade-offs	Gram positive	*T. halophilus*	Capsular polysaccharide (CPS)	Mutations in cps gene cluster (e.g., *wzy* ::IS) or *pgm*	Observed growth delay in specific mutants. Furthermore, the loss of CPS likely deprives the bacteria of a general protective barrier against environmental stresses.	(20)
		*C. perfringens*	Capsular polysaccharide (CPS)	CPS reduction (e.g., via *galE* mutation)	Theoretically increased susceptibility to immune clearance due to loss of anti-opsonization shield.	(21)
		*S. aureus*	Wall teichoic acid (WTA)	WTA deficiency or capsule overproduction	Diminished WTA-mediated adhesion to host endothelial cells and attenuated abscess formation.	(22)
			Unspecified	Mutations in global regulators (e.g., *mgrA, arlR* ), leading to loss of cell clumping ability.	Loss of physical barrier against neutrophil phagocytosis and reduced secretion of hemolytic toxins.	(25)
		*L. ivanovii*	Wall teichoic acid (WTA)	Loss of WTA glucose modifications	Abolished surface localization of virulence factor InlB, decreasing epithelial cell invasion efficiency.	(23)
		*E. faecalis*	Enterococcal polysaccharide antigen (Epa)	Epa deficiency or mutation	Attenuated adherence to intestinal epithelial cells and marked vulnerability to chemical barriers (bile salts, osmotic stress).	(45, 46)
	Gram negative	*A. bnaumanii*	Lipooligosaccharides (LOS)	Complete loss of LOS	Exposure of the underlying peptidoglycan layer, rendering the bacteria extremely vulnerable to rapid killing by neutrophil-secreted lysozyme.	(26)
			Longer lipooligosaccharides (LOS) and capsule	Loss of longer LOS and capsule	Increased C5b-9 deposition, membrane permeability, and marked serum killing.	(36)
		*P. gingivalis*	Capsule	Capsule loss or mutation	Loss of the anti-phagocytic shield and cell-specific immune modulation, disrupting its ability to suppress cytokines in fibroblasts while inducing them in dendritic cells.	(27–29)
			Lipopolysaccharide (O-antigen / Core)	Rough or semi-rough LPS (O-antigen truncation)	Increased susceptibility to serum complement, potent monocyte activation (TNF, IL-6), and impaired T3SS toxin (ExoU) injection.	(30, 31, 38)
		*P. aeruginosa*	Type IV pili (T4P)	Pili loss or mutation	Loss of twitching motility, stripping the physical capacity to adhere to and colonize host tissues, leading to decreased virulence.	(9, 43)
		*S. enterica*	Lipopolysaccharide (LPS)	LPS O-antigen truncation or deficiency	Loss of physical barrier, leading to increased susceptibility to contact-dependent interbacterial antagonism such as T6SS intoxication.	(32)
		*E. coli*	Lipopolysaccharide (LPS)	LPS modification or capsule overproduction (receptor masking)	Increased susceptibility to serum complement and heightened phagocytosis by macrophage models.	(33–35)
			Unspecified	Mucoid phenotype (via Rcs phosphorelay mutation)	Decreased motility, theoretically impairing dissemination capabilities within the host environment.	(44)
		*B. cenocepacia*	Lipopolysaccharide (LPS)	LPS truncation	Profound serum-mediated inactivation (susceptibility to complement).	(37)
		*K. pneumoniae*	Capsule (K1/K2 types)	Capsule loss (e.g., *wcaJ* mutation)	Stripped hypermucoviscous phenotype in certain mutants, causing loss of serum resistance and profound susceptibility to macrophage phagocytosis.	(39-41
Trade-ups	Gram positive	*S. pyogenes*	HA capsule	HA capsule loss	Increased intracellular survival by evading macrophage autophagy-dependent killing.	(54)
			Peptidoglycan	HA capsule overproduction as a barrier	Heightened resistance to host immune components, as the thick capsule blocks both phages and immune cells	(55)
	Gram negative	*P. aeruginosa*	Flagella	Flagella loss or motility defects	Acquisition of a stealth phenotype evading NETs, TLR5 recognition, and phagocytosis (via failure to induce PI3K/Akt phosphorylation), increasing *in vivo* pathogenicity.	(48–51)
		*E. coli*	Unspecified	Loss of fimbriae and flagella (*ΔfimAΔfliC* )	Significantly reduced monocyte phagocytosis, suggesting evasion from immune clearance.	(52)
			OmpC or Capsule	Loss of OmpC or capsule (Δ*ompC* or Δ*etp*)	Significantly increased serum survival compared to WT, facilitating evasion from complement-mediated killing.	(53)

## 3 Trade-offs as a Driver of Immune Clearance -Host Perspective

### 3.1 Unmasking the gram-positive outer wall: capsule and surface glycan shedding induces immunological vulnerability

Gram-positive capsules and cell wall polysaccharides act as formidable immune shields. For instance, the hyaluronic acid (HA) capsules of *Streptococcus pyogenes* prevent neutrophil-mediated killing (16), and capsule-deficient *Streptococcus pneumoniae* exhibit heightened susceptibility to macrophage and neutrophil phagocytosis (17). Similarly, *Staphylococcus aureus* coordinates its capsule with the Efb protein to evade phagocytosis across varying plasma concentrations (18). Loss of the poly-γ-D-glutamic acid capsule in *Bacillus anthracis* dramatically increases the binding of opsonins (C3b, IgG, CRP), accelerating phagocytosis (19). Thus, capsules are critical for *in vivo* survival. On the other hand, some phages exploit capsular polysaccharides (CPS) as primary receptors. For example, phages phiYG2_4 (targeting *Tetragenococcus halophilus*) and CPS1 (targeting *Clostridium perfringens*) use CPS for adsorption (20, 21). Consequently, acquiring phage resistance can entail suppressing CPS production. This could create an evolutionary dilemma: preserving this anti-opsonization shield invites phage lysis, while shedding it guarantees vulnerability.

Outer wall alterations cause diverse immunological vulnerabilities and virulence attenuation. In *Staphylococcus aureus*, wall teichoic acid (WTA) serves as both a critical colonization factor and the primary phage receptor. While mutating or deleting WTA to evade phages confers resistance, it forces a severe evolutionary trade-off by abolishing bacterial adhesion to host endothelial cells and significantly attenuating *in vivo* abscess formation (22). Alternatively, *S. aureus* can evade WTA-targeting phages by overproducing its capsular polysaccharide to physically mask the WTA receptors. Although this structural alteration effectively shields the bacteria from specific anti-WTA IgG deposition, it imposes a distinct fitness cost where the thickened capsule completely obstructs WTA-mediated adhesion to host cells, thereby severely compromising the colonization capacity of the pathogen (22). Loss of WTA glucose modifications, a known phage receptor in *Listeria ivanovii*, abolishes the surface localization of the virulence factor InlB, significantly impairing epithelial invasion (23). Furthermore, rhamnose-rich cell wall polysaccharides (Rha-CWPS), which are widely conserved among Gram-positive bacteria, serve as both phage receptors and targets for host lectin recognition. Consequently, it has been proposed that glycan mutations driven by phage evasion inevitably lead to altered immune recognition (24). In addition, according to a recent study (25), the acquisition of phage resistance in methicillin-resistant *S. aureus* (MRSA) is accompanied by mutations in global transcriptional regulators, such as *mgrA* and *arlR*, leading to a loss of cell clumping ability. Typically, MRSA evades neutrophil phagocytosis by binding to fibrinogen and forming protective aggregates. Phage-resistant mutants not only lose this physical barrier but also exhibit a simultaneous reduction in their capacity to secrete hemolytic toxins designed to destroy neutrophils. As a result, the MRSA strains that successfully escape phage predation are effectively disarmed, transforming into highly vulnerable targets for the host immune system.

### 3.2 Unmasking the gram-negative outer wall: LPS and capsule alterations induce immunological vulnerability

Gram-negative LPS and capsules act as formidable shields against neutrophils, macrophages, and complement. In *Acinetobacter baumannii*, lipooligosaccharide (LOS, often referred to as LPS) protects the peptidoglycan layer. Therefore, the complete loss of LOS renders the strain extremely vulnerable to neutrophil-secreted lysozyme, leading to rapid bacterial killing (26). Similarly, the *Porphyromonas gingivalis* capsule acts as a critical shield to evade phagocytosis and manipulates host inflammatory responses, thereby enhancing bacterial survival and virulence (27–29). Thus, mutating these structures to evade phages strips bacteria of defensive barriers, forcing a fatal evolutionary trade-off. In fact, truncating LPS O-antigen and core structures critically increases vulnerability to complement and immune clearance. Resistance against *Pseudomonas* phages often yields rough or semi-rough LPS, dramatically increasing serum complement susceptibility (30). Furthermore, O-antigen-deficient *P. aeruginosa* mutants (e.g., *wzy* or *wbpH*) show distinct 50% human serum susceptibility and strongly activate monocyte NF-κB, upregulating TNF and IL-6 to provoke potent inflammation (31). Beyond evasion of host immunity, O-antigen truncation imposes severe ecological trade-offs by eliminating the physical buffer between competing bacteria. When *Salmonella enterica* truncates its LPS O-antigen to resist phages, the diminished interbacterial distance renders the pathogen highly susceptible to contact-dependent killing via the type VI secretion system (T6SS) of neighboring *Enterobacter cloacae*. A similar elevation in T6SS vulnerability due to O-antigen truncation is also observed in *P. aeruginosa* (32).

Similar and unique phenomena occur across other Gram-negative bacteria. The probiotic *E. coli* Nissle 1917 naturally possesses a semi-rough LPS structure that confers resistance to T4 phages via direct inactivation while inherently maintaining high serum susceptibility (33, 34). In addition, *E. coli* clones undergoing LPS modification or capsule overproduction during phage resistance exhibit increased susceptibility to phagocytosis by *Dictyostelium discoideum*, a single-celled macrophage model (35). In multidrug-resistant *A. baumannii*, phage-resistant strains losing longer LOS and capsules display increased C5b-9 deposition, >70-fold higher membrane permeability, and marked serum killing (36). Phage-resistant *Burkholderia cenocepacia* with LPS mutations also suffer profound serum-mediated inactivation (37). Notably, it has been reported that phage resistance mutations in *wzzB* (O-antigen synthesis) or *wapH* (LPS core synthesis) in *P. aeruginosa* markedly reduce the secretion of type III secretion system (T3SS) effectors, such as ExoU (38). This indicates that the disrupted LPS fails to provide a critical surface scaffold, preventing proper T3SS assembly and function. Consequently, this loss of the toxin injection capability drastically attenuates the lethality of *Galleria mellonella*. Moreover, hypervirulent *Klebsiella pneumoniae* (hvKp) relies on thick capsules (e.g., K1/K2) for pathogenicity and immune evasion. Notably, the immunological trade-offs in phage-resistant *K. pneumoniae* are heterogeneous. While certain capsule-defective mutants become highly susceptible to serum killing and macrophage phagocytosis, others may even enhance their anti-phagocytic properties despite an overall reduction in virulence (39, 40). Specifically, mutations in *wcaJ*, a glycosyltransferase essential for capsule synthesis, completely strip hvKp of its hypermucoviscous phenotype and K1 capsular shield. In essence, the very moment the bacteria mutate *wcaJ* to escape the phage, they critically cripple their own defenses (41). On the other hand, it has been suggested that mutations in certain global regulatory systems such as *sdiA* in *K. pneumoniae* can simultaneously increase susceptibility to both phage infection and complement-mediated killing. This dual vulnerability may be linked to an elevated rate of bacterial filamentation, which exposes a larger cell surface area to both phage attachment and complement deposition (42).

### 3.3 Deprivation of motility and increased vulnerability to chemical barriers

Bacterial infection and colonization fundamentally rely on motility appendages (e.g., pili) and robust surface polymers to withstand harsh *in vivo* environments. Phage-driven selection actively targets these features, drastically accelerating pathogen clearance via the host’s physical and chemical barriers.

In *P. aeruginosa*, resistance to type IV pili (T4P)-targeting phages frequently induces mutations in pili biosynthesis genes (9). Consequently, bacteria lose their characteristic twitching motility, stripping them of the physical capacity to adhere and colonize tissues. *Galleria mellonella* models demonstrate that this loss of pili substantially reduces lethality, proving that phage-mediated selection attenuates virulence and severely compromises pathogenic potential (43). Similarly, in *E. coli*, mutations in the Rcs phosphorelay system confer mucoid phage resistance but simultaneously cause a significant decrease in motility. While the mucoid phenotype itself may enhance immune evasion, this severe loss of motility can critically impair the bacteria’s capacity for dispersal and dissemination within the host environment (44).

Furthermore, when phage resistance compromises surface glycans, pathogens become defenseless against severe chemical environments, such as the gastrointestinal tract. In *Enterococcus faecalis*, phage resistance often involves mutations in the enterococcal polysaccharide antigen (Epa) biosynthesis gene cluster. This Epa deficiency not only attenuates intestinal epithelial adherence but also dose-dependently increases susceptibility to bile salts (e.g., sodium deoxycholate). Consequently, murine oral infection models show significantly impaired intestinal colonization and a dramatic decrease in fecal bacterial counts (45). Additionally, loss of the *epaR* function in *E. faecalis* OG1RF reduces NPV1 phage adsorption to 1–2%, conferring robust resistance but imposing a fatal trade-off that includes extreme vulnerability to osmotic stress induced by high NaCl concentrations (46).

Ultimately, phage-driven surface modifications do more than just disrupt immune evasion. They drive pathogens into a powerless state that renders them unable to withstand the host’s network of physical and chemical barriers. This phenomenon represents another crucial pillar of immuno-phage synergy, ultimately dictating the *in vivo* elimination of bacteria.

## 4 Clinical implications & future directions

As discussed, converting phage resistance trade-offs into therapeutic gains requires a functional host immune system, highlighting an inherent limitation in immunocompromised patients. Even if bacteria shed defensive shields such as LPS or capsules to evade phages, the lack of effectors prevents their clearance, suggesting that localized sites of infection could be pathogen-dominated. Marchi et al.’s mathematical model proved that immuno-phage synergy requires the host’s innate immune capacity to exceed a specific threshold. Below this threshold, forcing bacterial trade-offs via monophage therapy is insufficient (47). However, their simulations revealed that rationally designed cocktails targeting completely independent receptors drastically reduce simultaneous double resistance, significantly improving outcomes. Thus, in environments lacking sufficient immune pressure, advanced cocktails laying multi-layered evolutionary traps are indispensable.

Loss of phage receptors does not universally guarantee a host-favorable trade-off; specific mutations risk inducing a “trade-up” that enhances immune evasion (**Table 1**). For instance, *P. aeruginosa* swimming motility robustly induces neutrophil extracellular traps (NETs). Consequently, mutants shedding flagella to escape phages lose colonization ability but may concurrently acquire a stealth phenotype evading neutrophil webs (48). In cystic fibrosis models, as *P. aeruginosa* flagella are critical toll-like receptor 5 (TLR5) targets, flagella-deficient strains exhibited increased pulmonary burdens and decreased survival, proving that flagellar absence impacts pathogenicity primarily via immune evasion rather than mere motility loss (49). *P. aeruginosa* clinical isolates also demonstrate that motility defects confer evasion from neutrophil recognition (50), fundamentally driven by the failure to induce initial PI3K/Akt phosphorylation in phagocytes (51). Similarly, a uropathogenic *E. coli* (UPEC) double mutant (*ΔfimAΔfliC*) showed significantly reduced monocyte phagocytosis (52). Such unexpected impacts highlight the severe risks of single-phage administration.

The divergence of evolutionary trajectories and trade-up risks are further demonstrated in enterohemorrhagic *E. coli* O157:H7. Comparing resistance against LPS/capsule-targeting phages versus OmpC-targeting phages reveals that the specific mutated receptor dictates entirely different adaptive cost profiles. While one route forces a dramatic reduction in virulence (trade-off), alternative routes of evasion risk maintaining advantageous traits (trade-up) (53). Additionally, capsule-deficient Group A *Streptococcus* exhibits increased intracellular survival by evading macrophage autophagy, illustrating that capsule loss does not always impose immunological costs (54). Conversely, if a capsule functions primarily as an overarching infection defense, as seen with the hyaluronic acid capsule of *S. pyogenes*, phage pressure might drive its overproduction, potentially selecting virulent strains with heightened immune resistance (55). Therefore, because host immune susceptibility varies drastically depending on the evolutionary trajectory, even against the same phage, strategically controlling the direction of evolution in phage therapy will be critical.

**Figure 1:**
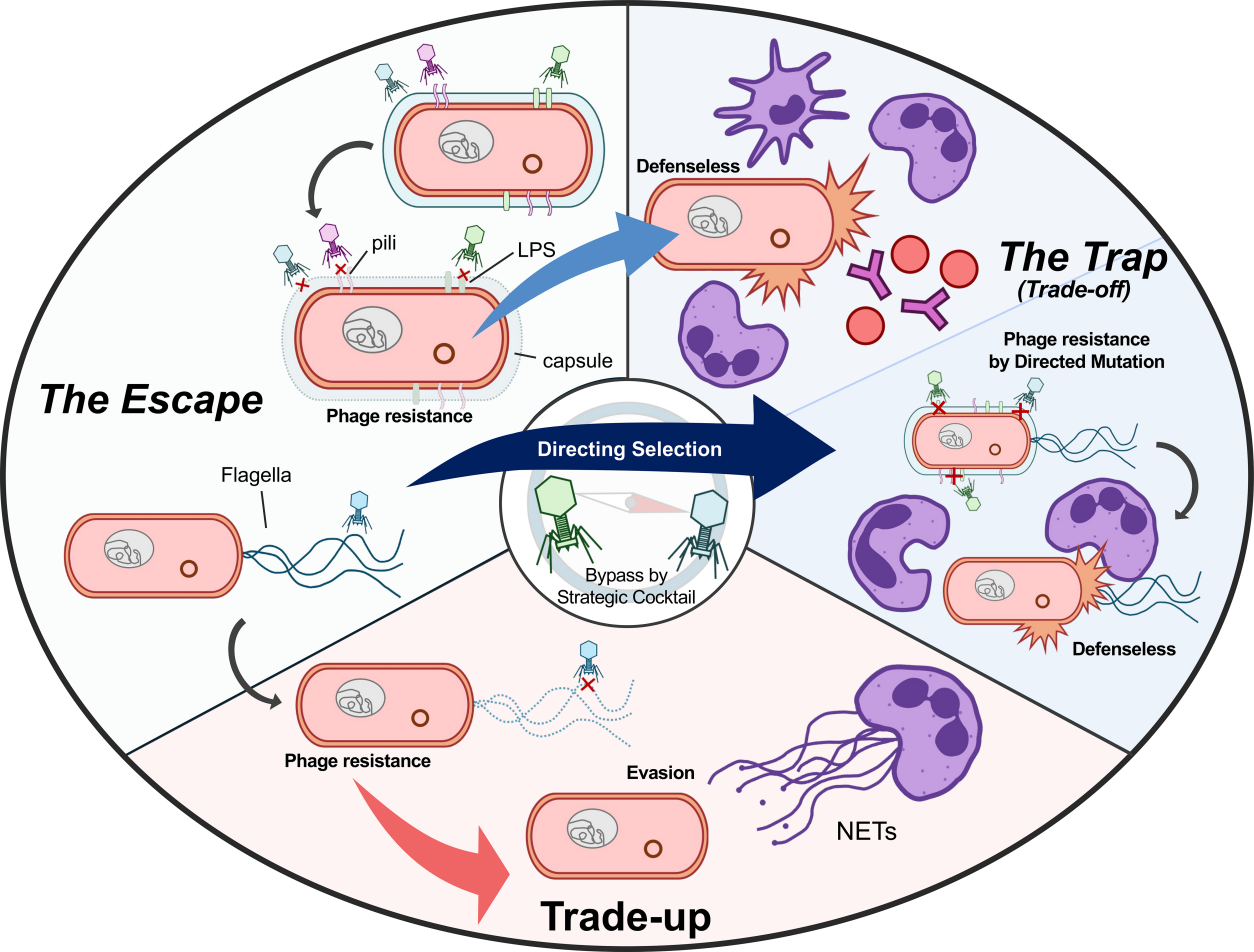
Conceptual framework for directing bacterial evolution in phage therapy: immunological synergy (trade-off traps) vs. evasion (trade-up risks). Bacterial adaptation to phage predation involves modifying essential surface structures that often double as virulence factors, leading to two contrasting evolutionary trajectories. In the desired scenario of immuno-phage synergy (Trade-off Traps), phage pressure forces bacteria to shed key defense barriers (e.g., capsules or LPS). This trade-off renders the pathogen highly vulnerable to innate immune clearance, such as complement-mediated killing and phagocytosis. Conversely, a counterproductive trajectory (Trade-up Risks) occurs when phage resistance inadvertently confers advantageous pathogenic traits, such as a “stealth” phenotype. For instance, the loss of immunogenic appendages like flagella can enable pathogens to evade immune detection (e.g., NETs). While standard phage therapy employs phage cocktails primarily to suppress the emergence of resistance, anticipating eventual resistance is crucial. Future therapeutic success may depend on exploring the design principles of advanced phage cocktails capable of bypassing evasive trade-ups and exclusively guiding bacteria into synergistic evolutionary traps.

These tripartite interactions highlight a significant paradox, the critical disconnect between *in vitro* evolution and *in vivo* reality. On standard media lacking immune cells, phage-resistant bacteria easily proliferate. *In vivo*, however, the intense selective pressure of the host immune system often clears variants with high immunological fitness costs prior to colonization. While *in vitro*-generated resistant bacteria have also emerged in *Galleria mellonella* models (43), further validation is crucial in humans and animals with advanced immune systems. Accurately assessing true clinical phage resistance risks requires next-generation *in vitro* infection models and platforms for selecting resistant variants directly *in vivo*, as previously discussed (56, 57). Based on these insights, future phage therapy should break away from passively asking “how to prevent resistance” and demand a proactive strategy that maps the bidirectional risks of immunological trade-offs and trade-ups. To guide evolution toward a desired trajectory, we could intentionally break spontaneous evolutionary selection by directing specific evolutionary paths. Ultimately, designing advanced phage cocktails that anticipate resistance endpoints and intentionally drive pathogens into “evolutionary traps” could become the next paradigm for sustainable clinical success (**Figure 1**).

## 5 Discussion and conclusion

The emergence of bacterial phage resistance has long been considered a primary obstacle to the clinical application of phage therapy. However, as explored in this mini-review, this formidable challenge can be fundamentally reframed as a unique therapeutic opportunity. When pathogenic bacteria mutate or shed essential surface structures such as capsules, LPS, or motility appendages to evade phage infection, they inadvertently strip away their primary defensive shields against the host immune system. This profound fitness cost, characterized by enhanced susceptibility to neutrophil-mediated killing, complement activation, and host chemical barriers, forms the mechanistic basis of immuno-phage synergy.

Recognizing this synergy allows us to shift the phage therapy from a reactive stance to a proactive one. Instead of engaging in an endless, unpredictable “evolutionary arms race” with bacterial pathogens, clinicians and researchers can leverage phage-induced selective pressures to intentionally drive bacteria into “evolutionary traps”, which were also discussed in previous reports (58, 59). By deeply understanding the concept of turning resistance into vulnerability, we can extract a significant therapeutic gain even when phage-resistant variants emerge in clinical settings. Indeed, it is highly plausible that such localized immuno-phage synergy has unknowingly contributed to the successful outcomes observed in previous clinical cases of phage therapy.

To fully realize this potential, the establishment of rational phage cocktail design principles that anticipate resistance trajectories is paramount. Future therapeutic strategies must focus not merely on preventing resistance, but on actively steering bacterial evolution toward clinically favorable, immune-sensitive phenotypes. Furthermore, coupling these immunological trade-offs with the well-documented phage resistance-induced antibiotic re-sensitization could yield an even more potent, multifaceted therapeutic strategy (4, 11–13, 25). This requires the integration of evolutionary biology and immunology to design advanced phage cocktails that predictably corner pathogens. Ultimately, embracing the dynamic interplay among phages, bacteria, and host immunity will be critical for maximizing the clinical efficacy and long-term sustainability of phage therapies in the future.
